# Identification and characterization of GRAS transcription factors in *Panax quinquefolius* and their potential roles in cold tolerance

**DOI:** 10.3389/fpls.2026.1859382

**Published:** 2026-05-20

**Authors:** Junmei Lian, Kairui Yao, Yifei Wang, Yingping Wang, Xiujuan Lei, Jian Zhang, Peng Di

**Affiliations:** 1 State Local Joint Engineering Research Center of Ginseng Breeding and Application, College of Chinese Medicinal Materials, Jilin Agricultural University, Changchun, China; 2 Faculty of Agronomy, Jilin Agricultural University, Changchun, China; 3 Department of Biology, University of British Columbia, Okanagan, Kelowna, BC, Canada

**Keywords:** cold stress, expression patterns, genome-wide analysis, GRAS, *Panax quinquefolius*

## Abstract

*Panax quinquefolius* L. is a perennial medicinal herb particularly vulnerable to recurring cold stress throughout its life cycle. The GRAS family constitutes a class of plant-specific transcription factors with pivotal functions in growth, development, and environmental adaptation. Nevertheless, a comprehensive characterization of the GRAS transcription factor family in *P. quinquefolius* remains lacking. A total of 123 PqGRAS members were identified and classified into 13 subfamilies. Transcriptomic profiling implicated that the PAT1 and DELLA subfamilies likely play pivotal roles in integrating multiple environmental and hormonal signals to coordinate complex stress-adaptive responses in *P. quinquefolius*. Notably, *PqGRAS086* (PAT1 subfamily) was significantly upregulated under both cold and methyl jasmonate (MeJA) treatments. Within the 2,000 bp upstream promoter of *PqGRAS086*, MeJA-responsive regulatory elements were enriched, whereas low-temperature-responsive elements were entirely absent. Phylogenetic analysis further revealed that *PqGRAS086* clustered with *VaPAT1*, *PtrPAT1*, and *ZjCIGR1*, which have been demonstrated to play a positive regulatory role in enhancing plant cold tolerance. RT-qPCR analysis further demonstrated that exogenous MeJA enhanced *PqGRAS086* expression during cold exposure, suggesting indirect involvement of *PqGRAS086* in cold stress through the JA signaling pathway. This study provides the first genome-wide characterization of GRAS genes in *P. quinquefolius* and identifies *PqGRAS086* as a candidate target for future functional studies and molecular breeding.

## 1 Introduction

*Panax quinquefolius* L. (American ginseng), a perennial herbaceous plant of the genus Panax in the family Araliaceae, is native to North America and was introduced to China in the 1980s. It is now cultivated across more than 35 countries worldwide (Sun et al., 2013; Lei et al., 2025). *P. quinquefolius* is rich in diverse bioactive compounds, including ginsenosides, polysaccharides, flavonoids, and amino acids, and has been widely used in medicine and health food products owing to its multiple pharmacological properties, such as immunomodulation, anti-fatigue, antioxidant, and antitumor activities (Hu et al., 2021; Zhao et al., 2025a). However, the escalating frequency of extreme weather events has rendered spring cold snaps an increasingly serious threat to *P. quinquefolius* production; low temperatures can injure buds, rhizomes, and seedlings, and in severe cases cause plant death, ultimately compromising both yield and quality. Furthermore, the relatively long growth cycle of *P. quinquefolius* exposes the plant to repeated cold stress challenges throughout its development. Therefore, identifying transcription factors (TFs) involved in regulating the cold stress response in *P. quinquefolius* may provide valuable insights for its molecular breeding and production.

TFs exert transcriptional control over target genes through sequence-specific interactions with genomic regulatory regions, thereby establishing complex gene regulatory hierarchies that are integral to plant growth, morphogenesis, and stress acclimation (Wu et al., 2022; Huo et al., 2025). Among them, the GRAS TF family is named after the three founding members GAI (Gibberellic Acid Insensitive), RGA (Repressor of GA), and SCR (Scarecrow) (Di Laurenzio et al., 1996; Peng et al., 1997; Silverstone et al., 1998). GRAS proteins span 400–800 amino acids, featuring a C-terminal GRAS domain of high sequence conservation alongside a structurally variable N-terminal region. The C-terminal domain contained five conserved structural elements arranged in a defined order: two leucine heptad repeats (LHRI and LHRII) flanking the central VHIID motif, followed by the PFYRE and SAW motifs (Hirsch and Oldroyd, 2009; Cenci and Rouard, 2017; Neves et al., 2023). Notably, DELLA proteins, a distinct clade within the GRAS family, harbor not only the conserved C-terminal GRAS domain (the gibberellin-signaling perception region) but also the DELLA and VHYNP domains in the N-terminal region, which constitute the GA repression region (Cui, 2025; Islam et al., 2025). The initial classification of the GRAS family into eight subfamilies (SCR, SCL3, LISCL, LAS, HAM, PAT1, DELLA, and SHR) was based on sequence characteristics of GRAS TFs first described in the model species *Arabidopsis thaliana* and *Oryza sativa* (Tian et al., 2004). The advent of high-throughput genome sequencing has facilitated genome-wide GRAS identification across diverse plant lineages, including *Solanum lycopersicum* (Huang et al., 2015), *Ananas comosus* (Lin et al., 2024), *Passiflora edulis* (Cai et al., 2025), and *Panax ginseng* (Wang et al., 2025b), with subfamily counts ranging from 8 to 17 depending on the species (Neves et al., 2023). Different clades have been shown to participate in distinct developmental processes. For example, DELLA proteins repress GA signaling while integrating endogenous hormonal and external environmental cues to fine-tune developmental plasticity (Davière and Achard, 2016; Zhao et al., 2025b). The SCR subfamily has been implicated in root development and leaf growth (An et al., 2025), whereas the LAS subfamily is considered essential for the formation of axillary shoot meristems (Greb et al., 2003; Feng et al., 2021).

GRAS TFs not only regulate plant growth and development but also play critical roles in multiple abiotic stress pathways, including responses to cold, drought, and salinity. In *Capsicum annuum*, *CaGRAS1* functions as a positive effector of drought tolerance, while the E3 ubiquitin ligase CaGIR1 acts antagonistically by destabilizing *CaGRAS1* (Oh et al., 2022; Baek et al., 2025). In *Populus davidiana* × *P. bolleana*, overexpression of the SCARECROW-LIKE (SCL) TF PdbSCL1 enhanced both drought tolerance and plant growth, whereas knockout lines exhibited reduced drought tolerance and growth (Wang et al., 2025a). In *S. lycopersicum*, overexpression of *SlGRAS4* significantly enhanced chilling tolerance in both leaves and fruits (Liu et al., 2020). In *A. thaliana*, *VaPAT1* overexpression conferred improved freezing tolerance in transgenic lines (Yuan et al., 2016). In *Vitis amurensis*, overexpression of *VaPAT1* enhanced the chilling tolerance of callus, suggesting that *VaPAT1* may function as a pivotal regulator of cold acclimation by steering JA biosynthetic flux under low-temperature conditions (Wang et al., 2021). In *Poncirus trifoliata*, cold stress induces *PtrPAT1*, which trans-activates *PtrBADH-l* by directly binding to its promoter, thereby promoting the accumulation of glycine betaine (GB). Furthermore, cold-induced *PtrPAT1* modulates the expression of *PtrPOD* and *PtrSOD*, facilitating ROS scavenging and ultimately enhancing cold tolerance (Ming et al., 2025). In *Zoysia japonica*, overexpression of *ZjCIGR1*, a PAT1 subfamily member, conferred enhanced cold stress tolerance, potentially through the regulation of downstream genes including *COR* genes (Kim et al., 2020). Despite accumulating evidence implicating GRAS TFs in abiotic stress adaptation, a genome-wide characterization of the GRAS family and its role in cold stress regulation in *P. quinquefolius* remains entirely lacking.

This study undertook a whole-genome level identification of GRAS TFs in *P. quinquefolius*, followed by comprehensive analyses of phylogenetic relationships, gene structure, conserved motifs, cis-regulatory elements, chromosomal distribution, collinearity, and expression profiles. Furthermore, based on integrated phylogenetic, expression pattern, and RT-qPCR analyses, a candidate *PqGRAS* gene, *PqGRAS086*, was identified as potentially involved in the cold stress response of *P. quinquefolius* through an indirect mechanism mediated by the jasmonic acid (JA) signaling pathway. Collectively, these analyses deepen our understanding of the structural, functional, and evolutionary characteristics of the PqGRAS gene family, and lay a foundation for future functional characterization of GRAS TFs in the cold stress response of *P. quinquefolius*.

## 2 Materials and methods

### 2.1 Identification of GRAS genes in *P. quinquefolius*

Genome assembly and annotation files for *P. quinquefolius* were retrieved from the National Genomics Data Center (NGDC Databases, https://ngdc.cncb.ac.cn/) under accession number GWHBEIR00000000.1 (Wang et al., 2022). *A. thaliana* GRAS protein sequences sourced from TAIR (https://www.arabidopsis.org/) were deployed as queries in a BLASTp search against the *P. quinquefolius* proteome via TBtools-II v2.388 (Chen et al., 2020). The HMM profile for the GRAS domain (PF03514) was downloaded from InterPro-EMBL-EBI (https://www.ebi.ac.uk/interpro/), and HMMER v3.2.1 was used to search for candidate GRAS sequences in the *P. quinquefolius* genome. In addition, the *P. quinquefolius* proteome was submitted to the transcription factor prediction module of PlantTFDB (https://planttfdb.gao-lab.org/) to predict GRAS TFs. Candidate PqGRAS proteins identified by the above three approaches were further validated using the SMART database (https://smart.embl.de/) and the Batch CD-Search module of the NCBI Conserved Domain Database (CDD) (https://www.ncbi.nlm.nih.gov/) to confirm the presence of the conserved GRAS domain, thereby finalizing the PqGRAS TFs list. The ExPASy ProtParam server (https://web.expasy.org/protparam/) was used to compute physicochemical parameters of PqGRAS proteins, including molecular weight (MW), theoretical isoelectric point (pI), instability index, aliphatic index, and grand average of hydropathicity (GRAVY) (Gasteiger et al., 2003). Subcellular compartment predictions were generated with WoLF PSORT (https://wolfpsort.hgc.jp/).

### 2.2 Phylogenetic analysis and classification of PqGRAS proteins

Reference GRAS sequences for *A. thaliana* and *O. sativa* were acquired from TAIR and Phytozome (https://phytozome.jgi.doe.gov/), respectively (Goodstein et al., 2012). A maximum likelihood phylogeny was inferred with IQ-TREE under the JTT+F+R7 substitution model, based on the combined dataset of 123 PqGRAS proteins from *P. quinquefolius*, 33 AtGRAS proteins from *A. thaliana*, and 50 OsGRAS proteins from *O. sativa*, with bootstrap support set to 1,000 replicates. Tree topology and branch annotations were displayed via Evolview (http://www.evolgenius.info/evolview-v2/). Four previously reported cold stress-related GRAS proteins were also included in the analysis: SlGRAS4 from *S. lycopersicum* (Liu et al., 2020), VaPAT1 from *V. amurensis* (Yuan et al., 2016), PtrPAT1 from *P. trifoliata* (Ming et al., 2025), and ZjCIGR1 from *Z. japonica* (Kim et al., 2020).

### 2.3 Gene distribution, duplication and collinearity analysis of *PqGRASs*

Chromosomal positions of *PqGRAS* genes were mapped using structural annotation data from the *P. quinquefolius* genome. Duplication events were detected with One Step MCScanX, and the outputs were rendered through the Gene Location Visualize (Advanced) and Advanced Circos modules embedded in TBtools-II v2.388 (Chen et al., 2022). Inter-species syntenic blocks connecting *P. quinquefolius* (Pq), *P. ginseng* (Pg), *Panax japonicus* (Pj), *A. thaliana* (At), and *O. sativa* (Os) were identified based on their respective genome assemblies and annotation files, with visualization performed in TBtools-II v2.388 (Chen et al., 2020). Reference genome data for *P. quinquefolius*, *P. ginseng*, and *P. japonicus* were downloaded from the NGDC Database; the *A. thaliana* assembly was retrieved from TAIR and the *O. sativa* assembly from Phytozome.

### 2.4 Gene structure, conserved motif and cis-regulatory elements analysis of *PqGRASs*

Conserved motif discovery for PqGRAS proteins was carried out using MEME Suite (https://meme-suite.org/meme/), with the motif count ceiling set at 10 and all remaining parameters at defaults. Domain composition was characterized through the Batch CD-Search module of the NCBI CDD, and the resultant Hitdata output was rendered in TBtools-II v2.388 (Chen et al., 2020). Promoter sequences spanning 2,000 bp upstream of each PqGRAS transcription start site were retrieved from the *P. quinquefolius* genome assembly and corresponding GFF3 file using TBtools-II v2.388 (Chen et al., 2020). CREs embedded within these promoter regions were annotated via the PlantCARE web tool (https://bioinformatics.psb.ugent.be/webtools/plantcare/html/), and the distribution of identified elements was displayed as a color-coded heatmap.

### 2.5 Three-dimensional structural prediction of PqGRAS proteins

Homology-based 3D structural modeling was conducted for all 123 PqGRAS proteins via SWISS-MODEL (https://swissmodel.expasy.org/). The optimal template for each protein was selected based on GMQE values and sequence identity scores, and the model best representing each subfamily was identified accordingly. Based on the structural and functional annotations of the selected templates provided by UniProt (https://www.uniprot.org/uniprotkb), including compositional bias, regions, domains, and motifs, the 3D structural models were visualized using PyMOL v3.1.

### 2.6 Expression profiling analysis of *PqGRASs*

Transcriptome data from root, leaf, and flower tissues of *P. quinquefolius* were generated by our laboratory in a previous study and are publicly accessible in the China National GeneBank Database (CNGBdb, https://db.cngb.org/) under Project ID CNP0001680 (Di et al., 2022). Transcriptome data from MeJA and abscisic acid (ABA) treatment experiments were obtained from previously published studies (Guo et al., 2024; Song et al., 2024). Plant material for cold treatment consisted of three-year-old *P. quinquefolius* transplant roots sourced from Ji’an, Tonghua, Jilin Province, China. Seedlings were exposed to 4 °C while control plants were maintained at 25 °C, and leaf samples were obtained at 0, 6, 8, 12, and 24 h. All tissue samples were immediately flash-frozen in liquid nitrogen and stored at −80 °C for transcriptome sequencing. A minimum of three biological replicates was maintained per treatment condition. Genes with no detectable expression at any time point (FPKM = 0) were excluded, and the remaining data were normalized row-wise using either the Zero-to-One or Normalized algorithm implemented in TBtools-II v2.388 (Chen et al., 2020). Genes responding to each of the three stress conditions were identified using R v4.3.1, with a threshold of *p* < 0.05; genes with FPKM < 1 at all time points were excluded from this analysis. Network Venn diagrams were generated using the online bioinformatics platform microbioinformatics (http://bioinformatics.com.cn/).

### 2.7 Plant materials and treatments

*P. quinquefolius* seeds were sourced from the State Local Joint Engineering Research Center for Ginseng Breeding and Application, Jilin Agricultural University. Uniformly germinated seeds were sown in nursery containers filled with a 1:1 (v/v) mixture of horticultural substrate and sand, and cultivated under standardized conditions (25 °C, 60% relative humidity, 16/8 h light/dark cycle) for 35 days prior to MeJA and cold treatments. For the treatment experiment, seedlings in the MeJA+Cold group were sprayed with 100 μmol/L MeJA at a fixed time each day until runoff, for two consecutive days, while the control group (CK+Cold) received an equal volume of distilled water. Leaf samples from both groups were collected at 0, 4, 8, 12, 24, and 48 h after the initiation of spraying. Subsequently, both groups were transferred to artificial climate incubators (Shanghai Santeng Instrument Co., Ltd., China) set at 4 °C, with all other growth conditions maintained as before, and leaf samples were again collected at 0, 4, 8, 12, 24, and 48 h. At every sampling point, a minimum of three independent samples were obtained, each consisting of leaves pooled from no fewer than three individual plants. All harvested material was immediately flash-frozen in liquid nitrogen and archived at −80 °C pending downstream analysis.

### 2.8 RNA extraction and RT-qPCR analysis

Total RNA was isolated from *P. quinquefolius* leaf tissue using the YALEPIC^®^ Plant Total RNA Fast Isolation Kit (PLUS). First-strand cDNA synthesis was carried out with the TransScript^®^ Uni All-in-One First-Strand cDNA Synthesis SuperMix for qPCR (One-Step gDNA Removal) (TransGen Biotech, Beijing, China). Quantitative RT-qPCR reactions were run on a Roche LightCycler 96 system (SYBR Green I, no passive reference dye) platform using SuperStar Universal SYBR Master Mix (CWBIO, Jiangsu, China). The thermocycling program consisted of an initial denaturation step at 95 °C for 30 s (1 cycle), followed by 45 cycles of denaturation at 95 °C for 10 s and combined annealing/extension at 60 °C for 20 s, with a final melting curve analysis from 95 °C for 15 s, 60 °C for 60 s, to 95 °C for 1 s. *PqGAPDH* (GenBank: MF614039.1) served as the endogenous reference gene for normalization (Song et al., 2024; Wang et al., 2025c), and transcript abundance was computed by the 2^-ΔΔCT^ algorithm. Detailed information on primer sequences used in this study is provided in **Supplementary Table 1**.

### 2.9 Statistical analysis

GraphPad Prism 10 software was used for statistical analysis, with one-way ANOVA for the assessment of the intergroup differences. At least three biological replicates were used for the calculation of standard deviation (± SD).

## 3 Results

### 3.1 Identification and characterization of *PqGRAS* genes

A total of 123 *PqGRAS* genes were identified through genome-wide screening of *P. quinquefolius*. Eight members unanchored to any chromosome were assigned the designations *PqGRAS001*–*PqGRAS008*, while the chromosomally positioned genes were sequentially named *PqGRAS009*–*PqGRAS123* (**Supplementary Table 2**). Physicochemical profiling via the ExPASy ProtParam server revealed that PqGRAS protein lengths spanned 313–795 amino acids (mean ≈ 549.22 aa), with molecular masses ranging from 36.34 kDa (PqGRAS064) to 87.14 kDa (PqGRAS096). Excluding PqGRAS044 and PqGRAS119, whose isoelectric point (pI) values exceeded 7, the remaining members exhibited pI below 7, consistent with an acidic character for the majority of the family. Instability index analysis indicated that 91.06% of PqGRAS proteins scored above 40, classifying them as intrinsically unstable, whereas the remaining 10 members fell below this threshold and are predicted to be relatively stable. Aliphatic index values ranged from 62.89 to 102.68 (mean ≈ 82.59), and GRAVY scores spanned −0.652 to 0.211; with the exception of PqGRAS012 and PqGRAS059, all members returned negative GRAVY values, reflecting an overall hydrophilic character. Subcellular localization prediction using WoLF PSORT indicated that 65% of PqGRAS proteins are preferentially nuclear (**Supplementary Table 2**).

### 3.2 Phylogenetic analysis of PqGRAS proteins

**Figure 1:**
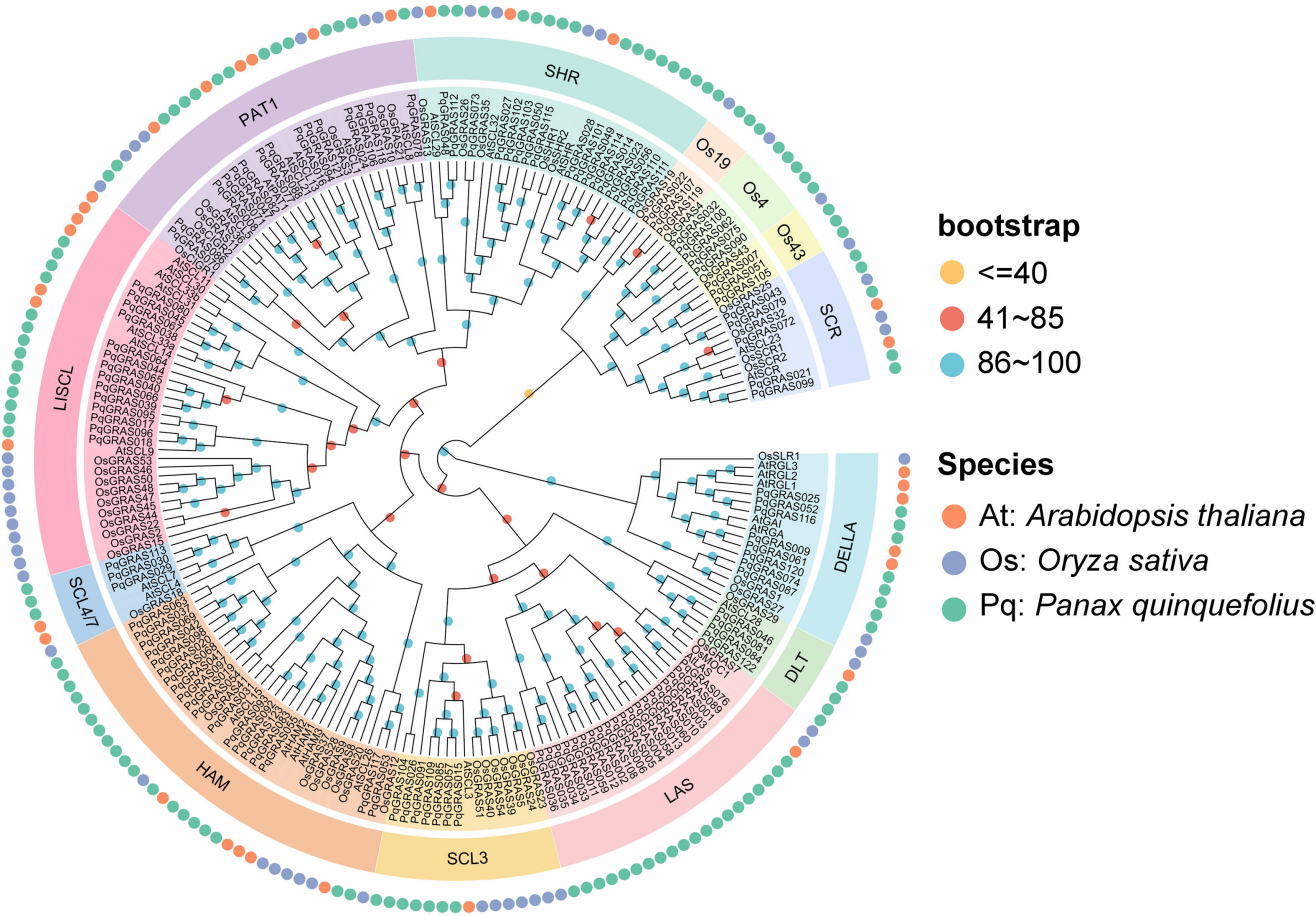
Unrooted phylogenetic tree of all GRAS proteins from *Panax quinquefolius*, *Arabidopsis thaliana* and *Oryza sativa*. PqGRAS proteins are divided into 13 groups indicated by different colors. The outermost orange, purple and green circle indicate the GRAS members from *A. thaliana*, *O. sativa* and *P. quinquefolius*, respectively.

To resolve evolutionary relationships among GRAS proteins from *P. quinquefolius* and other species, a maximum likelihood phylogeny was inferred from the combined dataset of 33 AtGRAS, 50 OsGRAS, and 123 PqGRAS sequences. Phylogenetic analysis assigned the PqGRAS proteins into 13 subfamilies: LAS, HAM, SHR, PAT1, LISCL, DELLA, SCL3, Os4, SCR, DLT, SCL4/7, Os19, and Os43 (**Figure 1**; **Supplementary Table 3**). The LAS subfamily was the largest, comprising 20 PqGRAS proteins, whereas SCL4/7, Os19, and Os43 were the least represented, each comprising only 3 proteins. The HAM subfamily was the second largest with 19 members, followed by SHR (17), PAT1 (15), and LISCL (14). In addition, the DELLA and SCL3 subfamilies contained 8 and 7 PqGRAS proteins, respectively. The DLT subfamily contained 4 members, and both the Os4 and SCR subfamilies each comprised 5 members.

Given the important roles of the PAT1 subfamily in plant cold stress responses, a focused phylogenetic analysis was performed by clustering the 15 P*. quinquefolius* PAT1 subfamily members together with four previously reported cold stress-related GRAS proteins to identify potential cold-tolerant candidate genes in *P. quinquefolius* (**Supplementary Figure 1**). The results showed that *PqGRAS070* and *PqGRAS086* clustered in the same clade as *VaPAT1*, *PtrPAT1*, and *ZjCIGR1*, all of which have been demonstrated to function as key positive regulators of cold tolerance in plants. In *V. amurensis*, *VaPAT1* activates *VaLOX3* transcription through a ternary complex with *VaIDD3* at the IDD-box, amplifying JA biosynthesis and bolstering cold tolerance (Wang et al., 2021). In *P. trifoliata*, cold-induced *PtrPAT1* drives *PtrBADH-l* expression via direct promoter occupancy, channeling carbon flux toward GB synthesis and enhancing freezing tolerance (Ming et al., 2025). In *Z. japonica*, *ZjCIGR1* overexpression fortified cold stress resilience, potentially by activating *COR* gene transcription (Kim et al., 2020). Collectively, these phylogenetic associations suggest that *PqGRAS070* and *PqGRAS086* may be functional participants in the cold stress regulatory network of *P. quinquefolius*.

### 3.3 Gene distribution, collinearity and evolution analysis of *PqGRASs*

**Figure 2:**
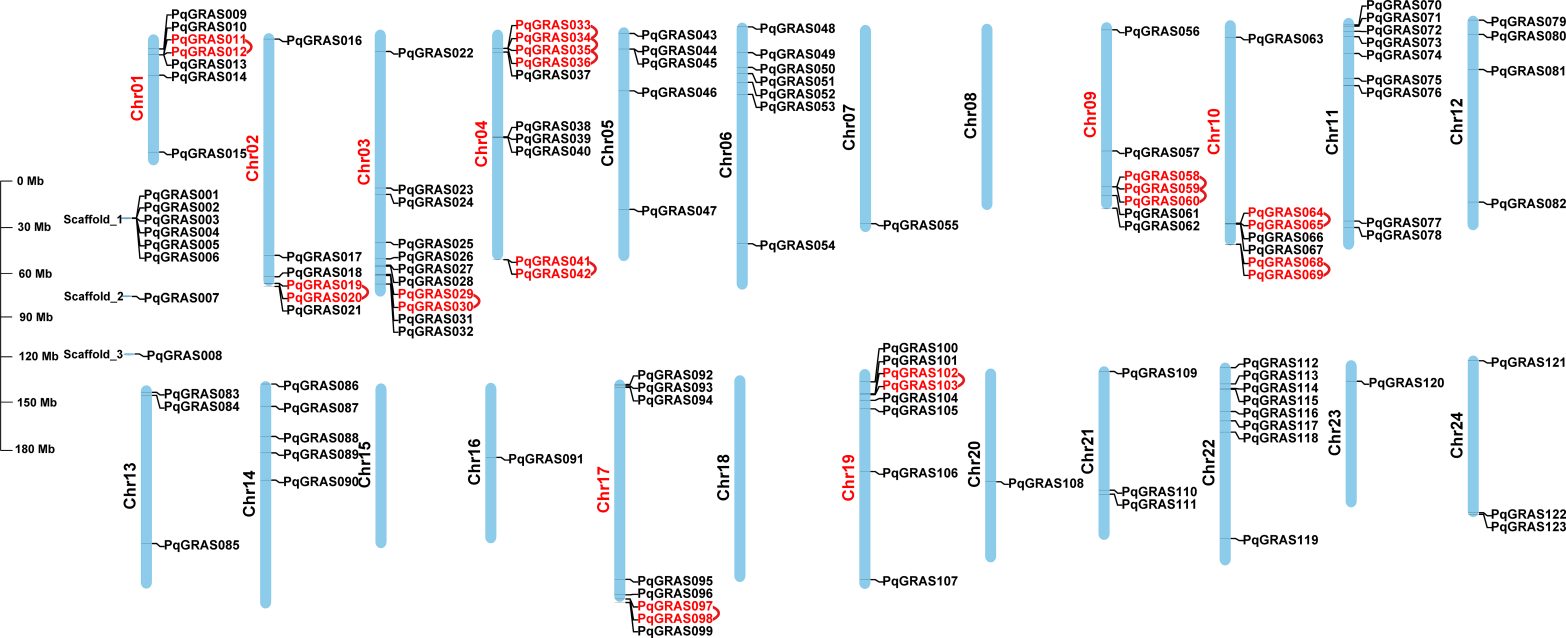
Gene distribution of 123 *PqGRAS* genes, with tandemly duplicated genes and chromosomes indicated in red. The unit of chromosome length is Mb.

**Figure 3:**
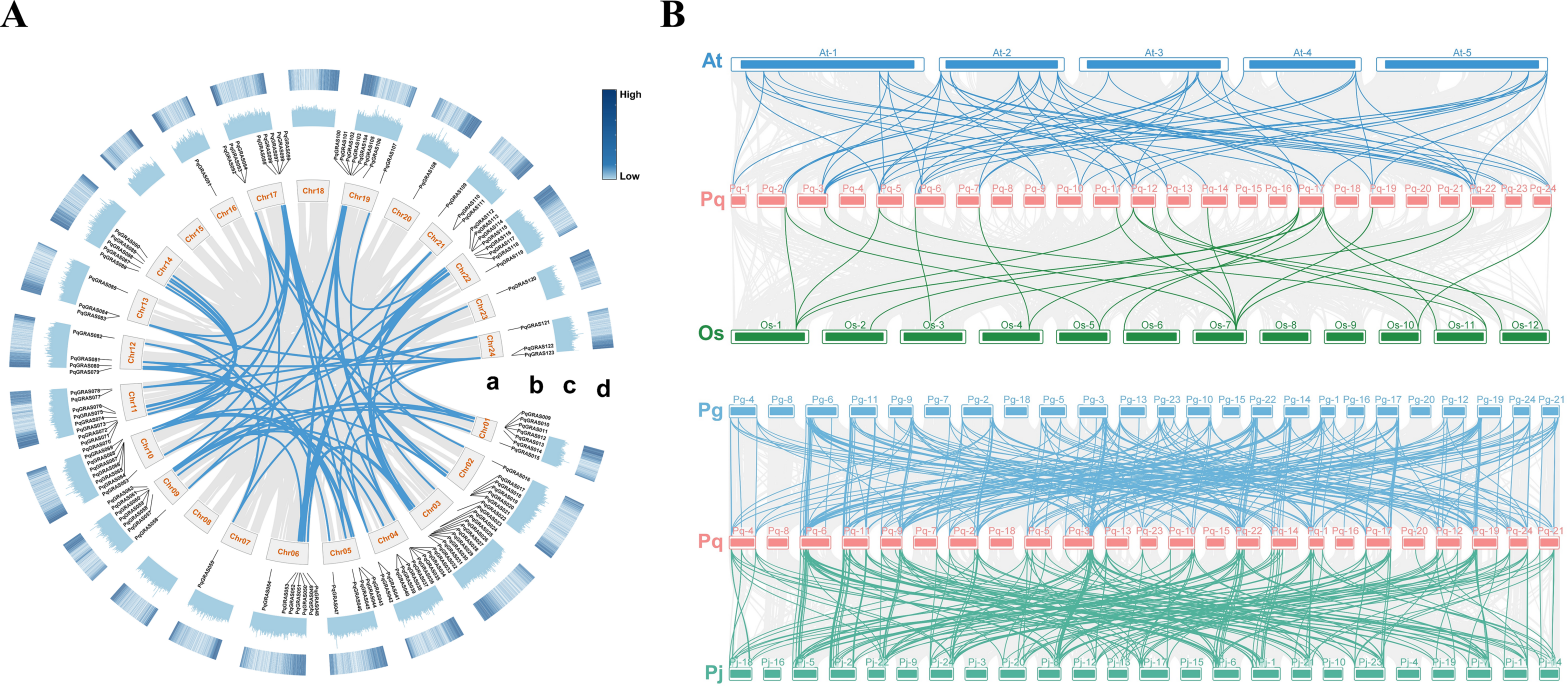
Gene collinearity and evolutionary analysis of *PqGRAS* genes. **(A)** Distribution and collinearity of *PqGRAS* genes in the *P. quinquefolius* genome (a: chromosome; b: gene ID; c: GC content; d: gene density). The background gray lines represent all the syntenic blocks in the *P. quinquefolius* genome, and the blue lines represent segment duplicate *PqGRAS* gene pairs. The blue color of the scale bar from light to dark represents increasing gene density. **(B)** Collinearity analysis of GRAS genes from *P. quinquefolius*, *A. thaliana*, *O. sativa*, *P. ginseng* and *P. japonius* (*Panax quinquefolius*: Pq; *Arabidopsis thaliana*: At; *Oryza sativa*: Os; *Panax ginseng*: Pg; *Panax japonius*: Pj). The 24 chromosomes of *P. quinquefolius* (Pq- 1-24), *P. ginseng* (Pg- 1-24) and *P. japonius* (Pj- 1-24), the 5 chromosomes of *A. thaliana* (At- 1-5), the 12 chromosomes of *O. sativa* (Os- 1-12) are depicted in different colors.

The distribution pattern of *PqGRAS* genes across the 24 chromosomes of *P. quinquefolius* was analyzed based on genome data. The 115 *PqGRAS* genes exhibited non−uniform distribution across 21 chromosomes, with the remaining 8 genes located on unassigned scaffolds; notably, Chromosome (Chr) 08, Chr15, and Chr18 harbored no *PqGRAS* genes (**Figure 2**; **Supplementary Table 2**). Chr03 harbored the greatest number of *PqGRAS* genes (11), followed by Chr04 (10) and Chr11 (9). Eight *PqGRAS* genes were distributed on each of Chr17, Chr19, and Chr22, and seven on each of Chr01, Chr06, Chr09, and Chr10, whereas Chr07, Chr16, Chr20, and Chr23 each contained only a single *PqGRAS* gene. The remaining chromosomes harbored between 3 and 6 *PqGRAS* genes each. To probe the mechanisms underlying PqGRAS family expansion, duplication events were detected via One Step MCScanX, identifing 13 tandem and 90 segmental duplication pairs (**Figures 2**, **3A**). Both duplication modes thus contribute to PqGRAS family expansion, with segmental duplication emerging as the dominant driver. Selective pressure acting on duplicated *PqGRAS* gene pairs was quantified by computing Ka/Ks (nonsynonymous/synonymous substitution) ratios in TBtools-II v2.388 (**Supplementary Table 4**) (Chen et al., 2020). As an indicator of selective pressure, Ka/Ks > 1 signals positive selection, suggesting potential acquisition of novel functions; Ka/Ks = 1 suggests neutral selection, with changes occurring randomly in the absence of positive selection; and Ka/Ks < 1 is diagnostic of purifying selection that maintains functional conservation (Kondrashov et al., 2002; Park et al., 2023). With the exception of one segmental duplication pair (*PqGRAS052*-*PqGRAS061*) for which no value could be calculated, values spanned 0.059–0.96 (mean ≈ 0.29). The Ka/Ks ratios of all calculated duplication pairs were < 1, suggesting that *PqGRAS* genes have predominantly undergone purifying selection during evolution, constraining the functional divergence of these duplicated gene pairs.

Inter-species collinearity was assessed by comparing *P. quinquefolius* with three dicotyledonous species (*A. thaliana*, *P. ginseng*, and *P. japonicus*) and one monocotyledonous species (*O. sativa*) to illuminate the evolutionary trajectory of *PqGRAS* genes (**Figure 3B**). A total of 102 *PqGRAS* genes showed collinear relationships with the *P. japonicus* genome, followed by *P. ginseng* (98), *A. thaliana* (38), and *O. sativa* (23) (**Supplementary Table 5**). These results indicate that *PqGRAS* genes share higher collinearity with congeneric species such as *P. ginseng* and *P. japonicus*, and that the degree of collinearity between *P. quinquefolius* and dicotyledonous species is greater than that between *P. quinquefolius* and the monocot *O. sativa*. Notably, 20 *PqGRAS* genes maintained synteny with all four representative species, implying that these genes have been functionally conserved throughout plant evolution (**Supplementary Table 5**).

### 3.4 Gene structure, conserved motif and domain analysis of PqGRAS proteins

**Figure 4:**
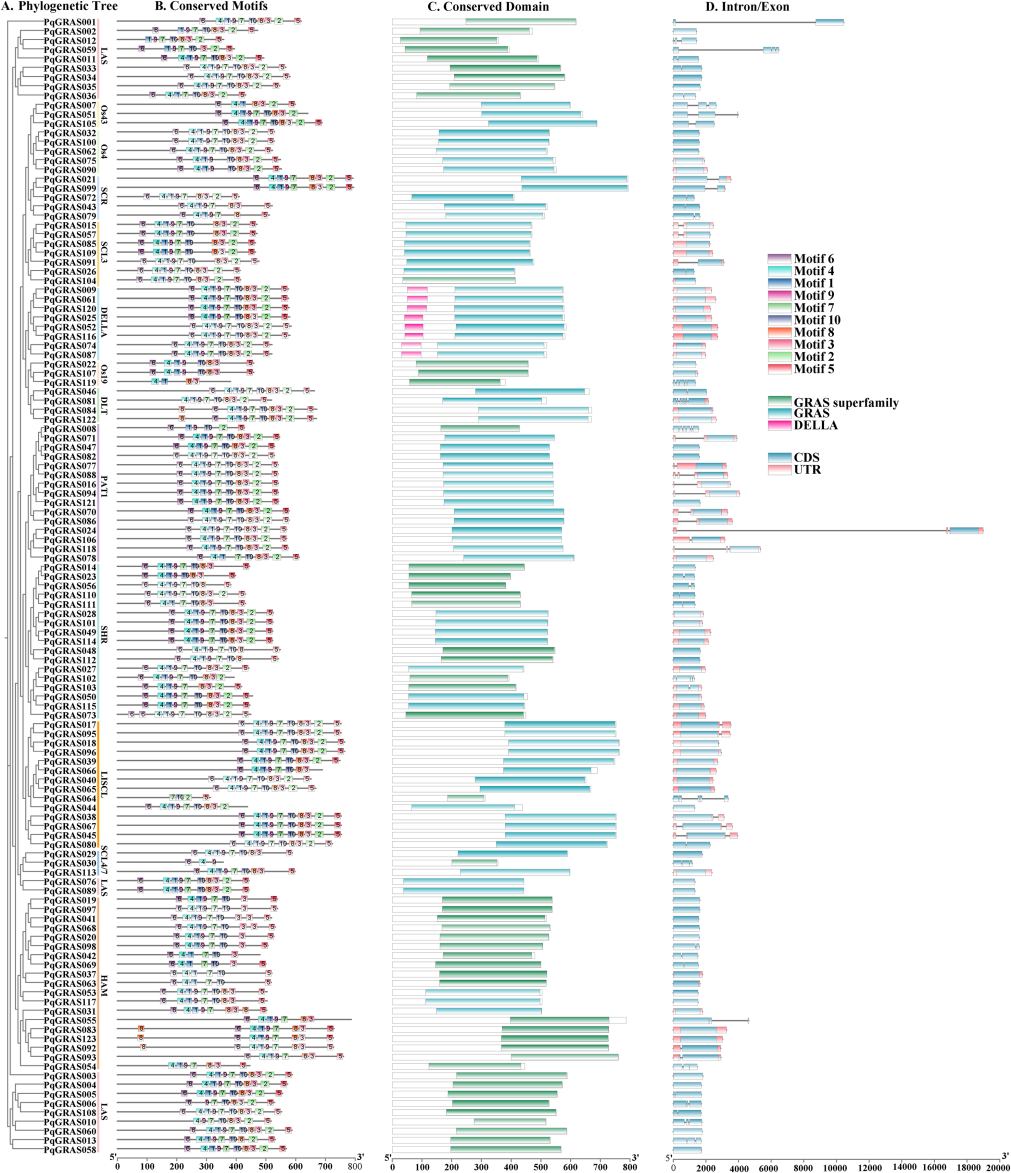
Phylogenetic relationships, conserved motifs, and gene structural analyses of GRAS proteins in *P. quinquefolius*. **(A)** Phylogenetic tree of PqGRAS proteins. **(B)** Distribution of conserved motifs of PqGRAS proteins, with motifs indicated by different colored boxes and gray lines indicating relative lengths of proteins. **(C)** Conserved domains of PqGRAS proteins, indicated by different colored rectangle. **(D)** Exon/intron structure of PqGRAS proteins. The UTR and CDS (exon) are represented by pink and blue boxes, introns is represented by black lines.

Structural characterization of PqGRAS proteins encompassed intron/exon architecture, conserved motif distribution, and domain composition, examined through MEME Suite and the NCBI CDD to assess structural diversity and its evolutionary implications (**Figure 4**). Structural features were displayed in accordance with the phylogenetic relationships of PqGRAS proteins (**Figure 4A**). Motifs 1, 4, and 6 were nearly ubiquitous across PqGRAS proteins (96.75%, 95.12%, and 95.12%, respectively), whereas the other motifs were detected only in a subset of proteins (**Figure 4B**). The SCL3 and DELLA subfamilies exhibited relatively stable and conserved domain compositions with no motif absences, while members of the other subfamilies showed varying degrees of motif loss, with the pattern of missing motifs differing among subfamilies. For example, all members of the SCL4/7 and HAM subfamilies lacked Motif 2, whereas all members of the SCR subfamily lacked Motif 10. All PqGRAS members contained either a GRAS domain or a GRAS Superfamily domain, and members of the DELLA subfamily additionally harbored a DELLA domain (**Figures 4B, C**). Intron/exon structure analysis revealed that 65 PqGRAS proteins (52.85%) lack introns entirely, while the remaining members contain between 1 and 6 introns (**Figure 4D**). Among these, PqGRAS081 of the DLT subfamily possessed the greatest number of introns. Within the same subfamily, PqGRAS proteins displayed similar structural characteristics. For instance, all SCR subfamily members contained a single intron, whereas all Os4 subfamily members were intronless. All DELLA subfamily members contained 10 motifs arranged in the order Motif 6, 4, 1, 9, 7, 10, 8, 3, 2, 5, and all possessed untranslated regions (UTR) with no introns.

### 3.5 Prediction of 3D structural modeling of PqGRAS proteins

**Figure 5:**
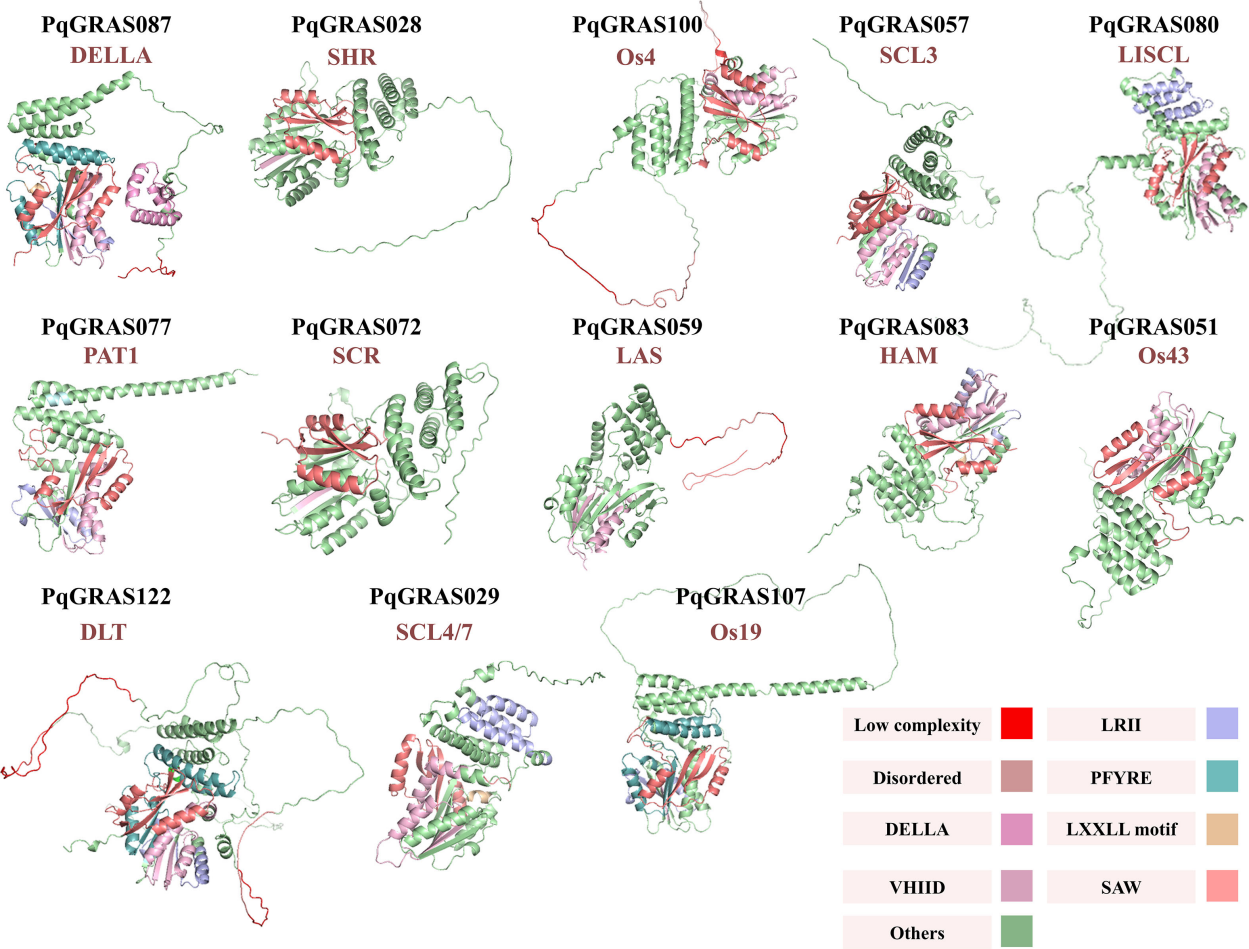
Predicted 3D structural modeling of PqGRAS proteins in *P. quinquefolius*. Different features of compositional bias, region, domain and motif are indicated by different colors. Low complexity (salmon), Disordered (red), DELLA (pink), VHIID (light pink), LRII (light blue), LXXLL motif (wheat), SAW (deep salmon).

The optimal structural template for each PqGRAS protein was selected using SWISS-MODEL for homology-based 3D model construction. The most representative model for each subfamily was then determined by integrating Global Model Quality Estimation (GMQE) values and sequence identity scores, and the models were visualized using PyMOL v3.1 based on domain annotations of the selected templates provided by UniProt (**Figure 5**; **Supplementary Table 6**). The 3D structural models revealed that all PqGRAS proteins share the conserved domain organization characteristic of the GRAS family, comprising an α-helical cap subdomain and an α/β core subdomain (**Figure 5**; **Supplementary Table 6**). The α/β core is centered on a β-sheet formed by multiple β-strands (typically 7-9), surrounded by the α-helical cap. The α-helical cap serves as one of the key active sites mediating protein-protein interactions, while the α/β subdomain, organized around the central β-sheet, not only maintains structural stability but also presents a series of functionally specialized variable grooves on its surface, which constitute an additional site for intermolecular interactions (Hakoshima, 2018). The majority of PqGRAS proteins contain 8 β-strands, whereas PqGRAS057, PqGRAS072, PqGRAS083, and PqGRAS087 each contain 9 β-strands. Taken together, the homology-based 3D structural analysis of PqGRAS proteins further confirms that they possess the conserved structural framework characteristic of the GRAS family, providing a basis for understanding their molecular functions.

### 3.6 Cis-regulatory element analysis of *PqGRAS* genes

**Figure 6:**
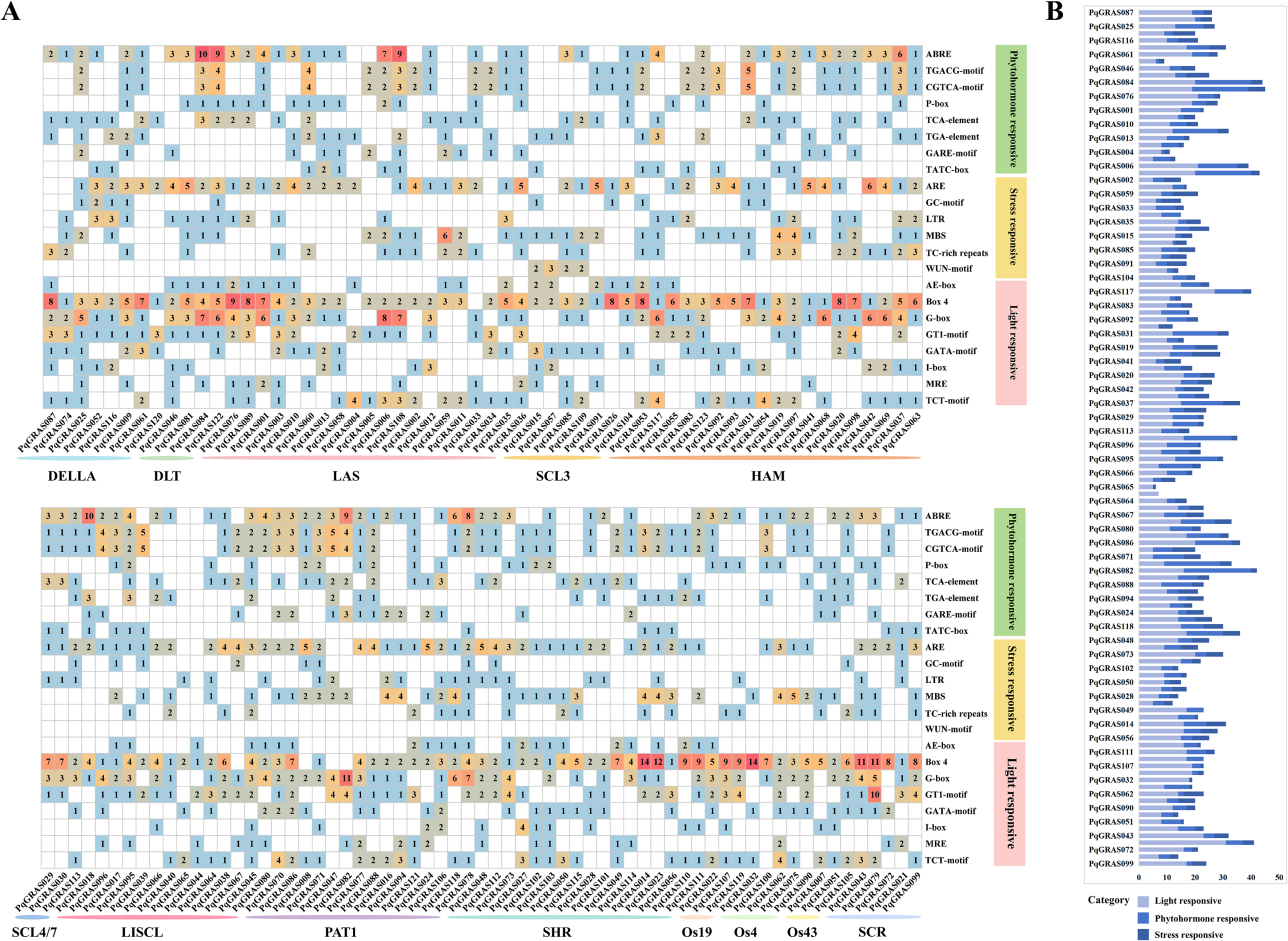
Number of cis-regulatory elements in the 2000 bp upstream promoter regions of *PqGRAS* genes. **(A)** Heatmap of the number of cis-regulatory elements, the different color presented the number of cis-elements. **(B)** The sum of cis-regulatory elements in each category is shown in the histogram.

CREs within the 2,000 bp upstream promoter regions of *PqGRAS* genes were annotated using PlantCARE and categorized into three functional classes: light-responsive (1,531; 54.23%), hormone-responsive (799; 28.30%), and stress-responsive (493; 17.46%). Hormone-responsive CREs comprised five categories: abscisic acid-, MeJA-, salicylic acid-, gibberellin-, and auxin-responsive CREs (**Figure 6A**). Among these, MeJA-responsive CREs represented the largest proportion (274; CGTCA-motif and TGACG-motif), followed by abscisic acid-responsive CREs (231; ABRE), gibberellin-responsive CREs (123; P-box, GARE-motif, and TATC-box), salicylic acid-responsive CREs (84; TCA-element), and auxin-responsive CREs (59; TGA-element). Stress-responsive CREs included anaerobic induction elements (ARE and GC-motif), drought-inducibility elements (MBS), defense and stress responsiveness elements (TC-rich repeats), low-temperature responsiveness elements (LTR), and wound-responsive elements (WUN-motif). Notably, light-responsive CREs were present in the promoter regions of all *PqGRAS* genes, with *PqGRAS079* of the SCR subfamily containing the greatest number (31), while *PqGRAS002*, *PqGRAS005*, *PqGRAS008*, *PqGRAS040*, *PqGRAS065*, *PqGRAS071*, and *PqGRAS101* each contained the fewest (5) (**Figure 6B**). Among light-responsive CREs, Box 4 (511, 33.38%), G-box (263, 17.18%), GT1-motif (150, 9.80%), and TCT-motif (120, 7.84%) were the most prevalent subtypes (**Figure 6A**).

### 3.7 Expression pattern analysis of *PqGRASs* in *P. quinquefolius*

**Figure 7:**
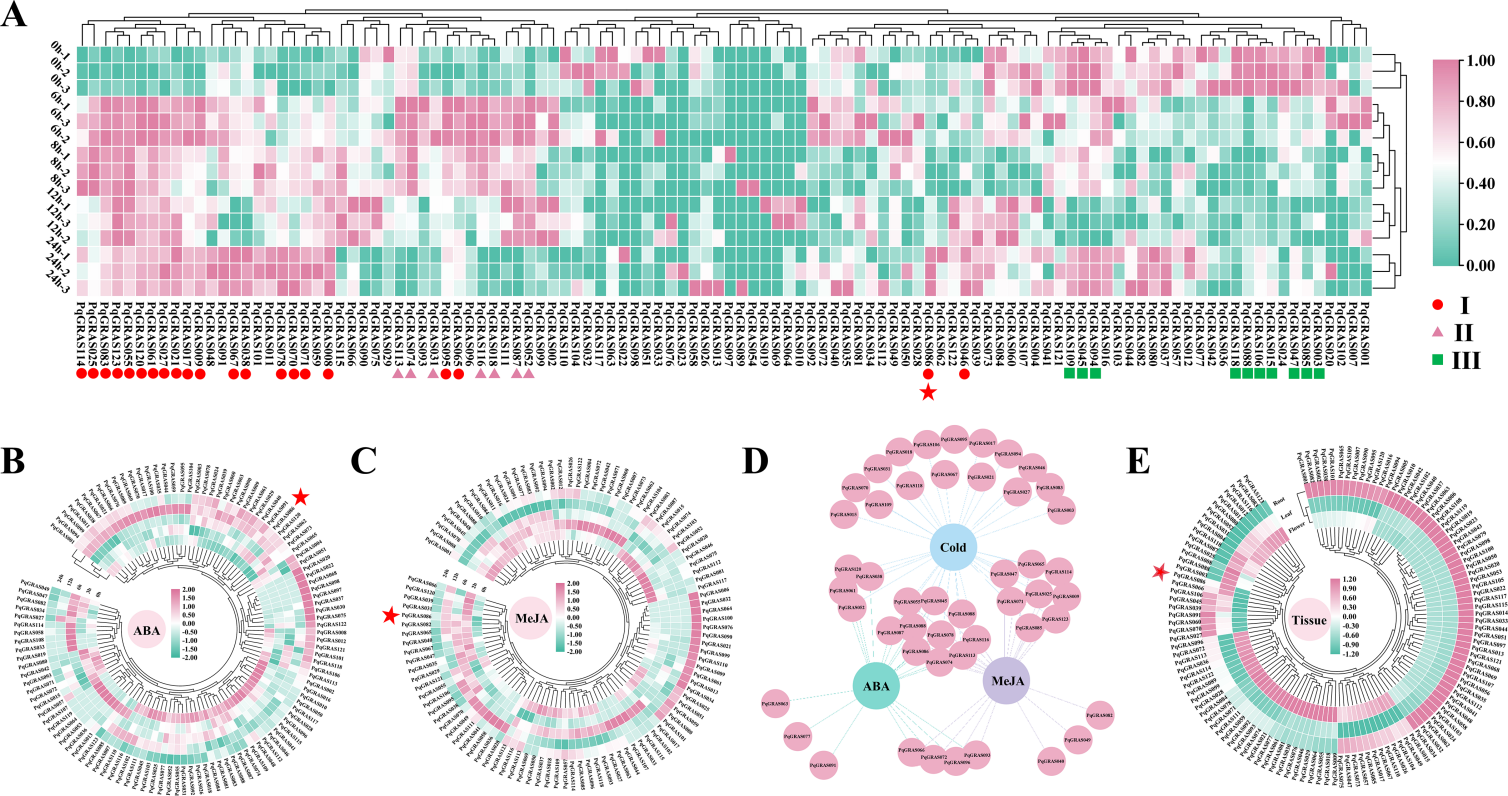
Expression pattern analysis of *PqGRAS* genes. **(A)** Expression patterns of *PqGRAS* genes under cold treatment. **(B)** Expression patterns of *PqGRAS* genes under ABA treatment. **(C)** Expression patterns of *PqGRAS* genes under MeJA treatment. **(D)** network Venn diagram of *PqGRAS* genes involved in responses to ABA, MeJA, and cold treatments. **(E)** Expression patterns of *PqGRAS* genes across diverse tissues (Root, Leaf and Flower). Expression levels under cold treatment were normalized by row using the zero-to-one algorithm, whereas expression levels under ABA and MeJA treatments and across different tissues were normalized by row using the normalized algorithm. The color scale ranging from green to pink indicates expression levels from low to high.

Given that GRAS TFs are not only widely involved in abiotic stress responses but are also closely associated with multiple phytohormone signaling pathways (Wang et al., 2021; Jaiswal et al., 2022; Neves et al., 2023), the expression profiles of *PqGRAS* genes under cold, ABA and MeJA treatments were further analyzed to systematically evaluate their potential roles in stress responses and hormonal regulation. Genes showing statistically significant expression changes (*p* < 0.05) at any time point relative to 0 h were considered responsive, and genes with FPKM < 1 at all time points were excluded from the analysis. A total of 38 *PqGRAS* genes were responsive to cold stress and were classified into three groups: group I (21 genes), group II (7 genes), and group III (10 genes), based on their expression patterns (**Figure 7A**; **Supplementary Table 7**). *PqGRAS* genes of group I showed upregulated expression at 6 h compared to 0 h and maintained elevated expression levels at 24 h. *PqGRAS* genes of group II also showed increased expression at 6 h, but their expression declined to levels at or below those at 0 h by 24 h. *PqGRAS* genes of group III were downregulated under cold stress. Within group I, *PqGRAS009*, *PqGRAS017*, *PqGRAS027*, *PqGRAS061*, *PqGRAS065*, *PqGRAS083*, *PqGRAS095*, and *PqGRAS120* peaked at 6 h, while *PqGRAS025*, *PqGRAS055*, and *PqGRAS114* peaked at 8 h, and the remaining group I genes reached their maximum expression at 24 h. Notably, although expression levels of most group I genes fluctuated across time points, only *PqGRAS008* and *PqGRAS078* showed a continuous and progressive increase over time. At 24 h, most group I genes showed expression levels 1- to 2.5-fold of those at 0 h, while *PqGRAS008*, *PqGRAS021*, and *PqGRAS086* exhibited expression levels approximately 3-fold of those at 0 h. All group II genes peaked at 6 h, followed by a decline in expression at 8 h. Group III genes were downregulated under cold stress, with 5 members (*PqGRAS047*, *PqGRAS088*, *PqGRAS094*, *PqGRAS106*, and *PqGRAS118*) belonging to the PAT1 subfamily. Notably, *PqGRAS088* of the PAT1 subfamily had the highest basal expression level (FPKM = 76.68 at 0 h) and exhibited the greatest magnitude of downregulation (0 h/6 h ratio > 4). The cold stress-responsive genes spanned 10 subfamilies, with the PAT1 subfamily contributing the most members (10), followed by the DELLA subfamily (8) and the LISCL subfamily (7).

A total of 22 *PqGRAS* genes were responsive to ABA treatment (**Figure 7B**; **Supplementary Table 7**). Among these, *PqGRAS061*, *PqGRAS063*, *PqGRAS066*, *PqGRAS086*, *PqGRAS091*, and *PqGRAS120* showed elevated expression at 3 h that remained above 0 h levels at 24 h. *PqGRAS074* showed no appreciable change at 3 h but underwent a sharp decline at 6 h, while the remaining ABA-responsive genes showed decreased expression at 3 h. Notably, *PqGRAS087* of the DELLA subfamily was the only gene to show a continuous and progressive decline in expression over time. A total of 25 *PqGRAS* genes were responsive to MeJA treatment (**Figure 7C**; **Supplementary Table 7**). *PqGRAS040*, *PqGRAS047*, *PqGRAS065*, *PqGRAS066*, *PqGRAS071*, *PqGRAS072*, *PqGRAS082*, and *PqGRAS086* were upregulated at 2 h relative to 0 h (2 h/0 h ratio > 1.5). Among these, *PqGRAS040*, *PqGRAS047*, *PqGRAS071*, and *PqGRAS072* showed transient upregulation at 2 h followed by rapid decline, whereas *PqGRAS086* maintained consistently elevated expression above 0 h levels across all time points despite some fluctuation, suggesting that it may play a central role in sustaining the defense state. The remaining 14 MeJA-responsive genes showed decreased expression at 2 h. Fourteen *PqGRAS* genes were co-responsive to both ABA and cold stress, 18 were co-responsive to both MeJA and cold stress, and 10 responded to all three treatments (**Figure 7D**; **Supplementary Figure 2**). The genes responsive to all three treatments were predominantly enriched in the PAT1 (*PqGRAS008*, *PqGRAS078*, *PqGRAS086*, and *PqGRAS088*) and DELLA (*PqGRAS074*, *PqGRAS087*, and *PqGRAS116*) subfamilies, suggesting that these two subfamilies may play pivotal roles in integrating multiple environmental and hormonal signals to coordinate complex stress responses in *P. quinquefolius*. Further analysis revealed that *PqGRAS086* (PAT1 subfamily) was upregulated under all three treatments (cold, ABA and MeJA), suggesting that its involvement in the cold stress response of *P. quinquefolius* may be associated with hormonal signaling pathways (**Supplementary Figure 2**).

To investigate the potential biological functions of *PqGRAS* genes, their expression profiles across three key tissues (flower, leaf, and root) were analyzed using RNA-seq data (**Figure 7E**; **Supplementary Table 7**). Except for *PqGRAS054*, which showed no detectable expression in any of the three tissues (FPKM = 0), the remaining *PqGRAS* genes displayed diverse expression patterns. Five genes (*PqGRAS067*, *PqGRAS078*, *PqGRAS086*, *PqGRAS113*, and *PqGRAS120*) were identified as constitutively highly expressed, exhibiting consistently high and balanced expression across all three tissues (FPKM > 10), suggesting potential roles in the growth and development of *P. quinquefolius*. Several genes displayed tissue-preferential expression patterns. For example, *PqGRAS024* showed preferential expression in root (FPKM = 13.44), with expression levels 3–4-fold higher than in leaf and flower. *PqGRAS009*, *PqGRAS055*, *PqGRAS061*, *PqGRAS092*, and *PqGRAS093* were preferentially expressed in flower; among these, *PqGRAS092* and *PqGRAS093* showed relatively high expression in flower (FPKM > 10) but low expression in leaf and root (FPKM < 2.5), whereas *PqGRAS009*, *PqGRAS055*, and *PqGRAS061* exhibited high expression in flower (FPKM: 21.68–89.92) while maintaining moderate expression levels in leaf and root (FPKM > 8), with flower expression levels 2–5 folds higher than in other tissues. These differential expression patterns suggest that *PqGRAS024* may play a predominant role in root development, *PqGRAS092* and *PqGRAS093* may be involved in regulating the specialized development of floral organs, and *PqGRAS009*, *PqGRAS055*, and *PqGRAS061* may participate in integrating flowering signals with overall plant growth and development. In addition, 35 *PqGRAS* genes showed very low expression levels across all three tissues (FPKM < 1), which may be attributable to spatiotemporal differences in their expression patterns.

### 3.8 RT-qPCR analysis

**Figure 8:**
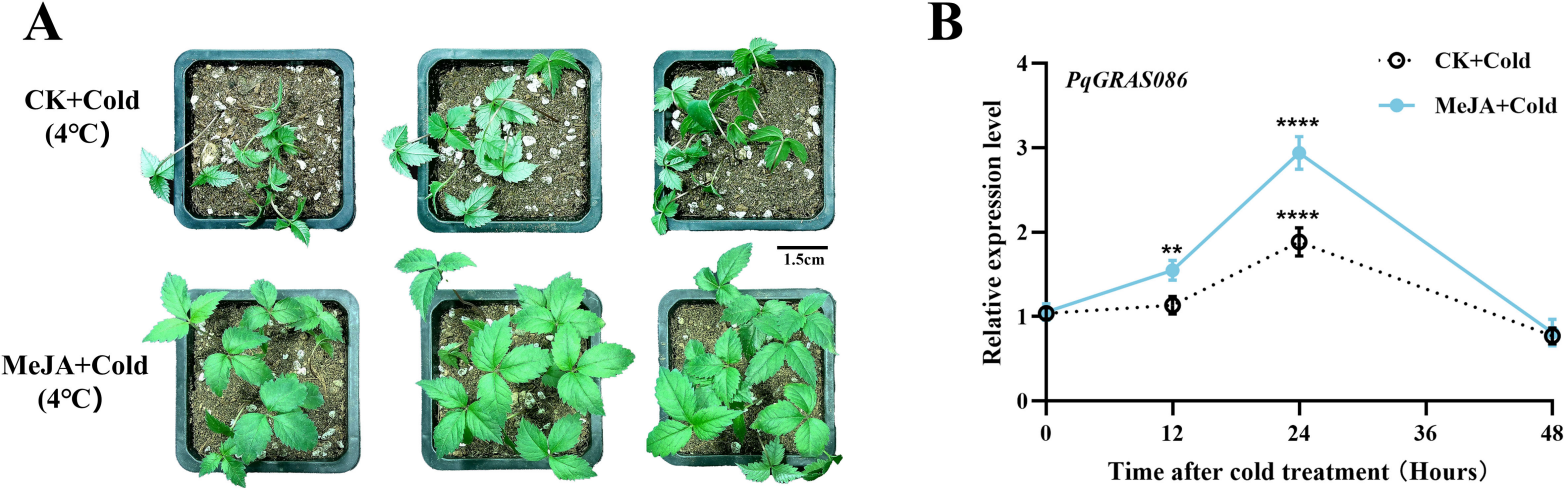
MeJA treatment enhanced cold tolerance in *P. quinquefolius* seedlings. **(A)** Morphology of *P. quinquefolius* seedlings treated with MeJA (MeJA+Cold) or without MeJA (CK+Cold) after storage at 4 °C for 48 h. **(B)** Relative expression levels of *PqGRAS086* in *P. quinquefolius* seedlings with or without exogenous MeJA treatment during cold stress (4 °C) were determined by RT-qPCR. Error bars represent the standard deviation of three biological replicates (**p < 0.01, ****p < 0.0001).

Given that *PqGRAS086* clusters in the same phylogenetic clade as VaPAT1, a TF known to integrate JA signaling with the cold stress response, and that *PqGRAS086* is transcriptionally induced by both MeJA and cold stress, we hypothesized that the involvement of *PqGRAS086* in the cold stress response of *P. quinquefolius* may be mediated through the JA signaling pathway (**Supplementary Figure 1**) (Wang et al., 2021). To test this hypothesis, 35-day-old *P. quinquefolius* seedlings were used as experimental material, and RT-qPCR was performed to compare the expression dynamics of *PqGRAS086* between seedlings subjected to MeJA pretreatment followed by cold stress (MeJA+Cold) and those sprayed with an equal volume of distilled water followed by cold stress (CK+Cold). Under normal temperature conditions (25°C), no obvious phenotypic differences were observed between the MeJA-treated and CK groups, suggesting that exogenous MeJA application did not affect the overall growth status of the seedlings under non-stress conditions (**Supplementary Figure 3**). In contrast, compared with the CK+Cold group, MeJA-pretreated seedlings exhibited less leaf wilting after cold stress exposure, indicating that exogenous MeJA treatment enhanced cold tolerance in *P. quinquefolius* seedlings (**Figure 8A**). RT-qPCR analysis further showed that MeJA pretreatment significantly elevated the expression level of *PqGRAS086* at 24 h of cold stress compared with the CK+Cold group (**Figure 8B**). These results collectively suggest that MeJA pretreatment may prime *PqGRAS086* expression to enhance cold stress tolerance in *P. quinquefolius* seedlings.

## 4 Discussion

GRAS TFs, as a class of plant-specific key regulatory proteins, play important roles in multiple aspects of plant biology, including growth and development, hormone signal transduction, and abiotic stress responses (Liu et al., 2021b; Neves et al., 2023). As a perennial medicinal plant with a long growth cycle and high economic value, *P. quinquefolius* is inevitably exposed to various abiotic stresses throughout its life cycle, among which low temperature is one of the key factors limiting its yield and quality. Therefore, mining gene resources associated with the cold stress response in *P. quinquefolius* is of great significance for its molecular breeding. In the present study, GRAS TFs in *P. quinquefolius* were systematically identified, and their phylogenetic classification, chromosomal distribution, and gene structure were comprehensively characterized. Furthermore, the expression patterns of PqGRAS TFs across different tissues (root, flower, and leaf) and under three treatments (cold, ABA, and MeJA) were analyzed based on transcriptome data, leading to the identification of several candidate genes potentially playing key roles in cold stress responses and hormonal signaling. These findings provide a foundation for future functional validation studies on the roles of PqGRAS TFs in the cold stress response of *P. quinquefolius* and offer potential targets for molecular breeding.

In the present study, 123 *PqGRAS* genes were identified from the *P. quinquefolius* genome, a number greater than that reported in *A. thaliana* (33) (Tian et al., 2004), *Paeonia ludlowii* (45) (Sun and Yuan, 2025), and *Perilla frutescens* (83) (Lian et al., 2026), but fewer than in *P. ginseng* (139) (Wang et al., 2025b) and *Gossypium hirsutum* (150) (Zhang et al., 2018). Interestingly, despite *P. quinquefolius* having a larger genome (3.57 Gb, 123) than both *P. ginseng* (3.36 Gb, 139) and *G. hirsutum* (2.31 Gb, 150), it harbors fewer GRAS genes than either species (Zhang et al., 2015, 2018; Wang et al., 2022, 2025b), suggesting that the size of the GRAS gene family is not strictly correlated with genome size. The 123 *PqGRAS* genes are unevenly distributed across 21 chromosomes (with Chr08, Chr15, and Chr18 containing no *PqGRAS* genes) and 3 scaffolds, and were designated *PqGRAS001*-*PqGRAS123* based on their chromosomal positions (**Supplementary Table 2**). Phylogenetic analysis classified GRAS proteins from *P. quinquefolius*, *A. thaliana*, and *O. sativa* into 13 subfamilies, with the LAS subfamily being the largest (20) and the HAM subfamily the second largest (19) (**Figure 1**; **Supplementary Table 3**). Gene duplication analysis identified 13 tandem duplication events and 90 segmental duplication events (**Figure 2** and **Figure 3A**; **Supplementary Table 4**). Segmental duplication appears to play a more predominant role in the expansion of the GRAS gene family in *P. quinquefolius*, in contrast to *Dimocarpus longan*, where segmental (8) and tandem (8) duplications occurred at equal frequencies, indicating that the mechanisms driving GRAS family expansion differ across lineages (Cao et al., 2025). Furthermore, among the 13 tandem duplication pairs, 5 pairs belong to the LAS subfamily (*PqGRAS033*-*PqGRAS034*, *PqGRAS034*-*PqGRAS035*, *PqGRAS035*-*PqGRAS036*, *PqGRAS058*-*PqGRAS059*, and *PqGRAS059*-*PqGRAS060*) and 4 pairs to the HAM subfamily (*PqGRAS019*-*PqGRAS020*, *PqGRAS041*-*PqGRAS042*, *PqGRAS068*-*PqGRAS069*, and *PqGRAS102*-*PqGRAS103*), which may partly account for the relatively large number of members in these two subfamilies. Collinearity analysis between *P. quinquefolius* and *A. thaliana*, *O. sativa*, *P. ginseng*, and *P. japonicus* revealed higher collinearity between *P. quinquefolius* and dicotyledonous species. Moreover, 102 and 98 *PqGRAS* genes showed collinear relationships with the *P. japonicus* and *P. ginseng* genomes, respectively, suggesting that the GRAS regulatory network may be highly functionally conserved at the genus level in Panax (**Figure 3B**; **Supplementary Table 5**).

Consistent with previous studies, all PqGRAS proteins were found to contain either a GRAS domain or a GRAS Superfamily domain, and members of the DELLA subfamily additionally harbored a DELLA domain (**Figure 4C**) (Tian et al., 2004). Intron-exon structure analysis revealed that 52.85% of *PqGRAS* genes (65/123) lack introns (**Figure 4D**), a proportion comparable to that observed in *D. longan* (55.40%, 31/56), *Dactylis glomerata* (56.52%, 26/46), and *A. comosus* (60.50%, 26/43), but lower than in *P. frutescens* (85.54%, 71/83) and *P. edulis* (93.10%, 27/29) (Zeng et al., 2019; Lin et al., 2024; Cai et al., 2025; Cao et al., 2025; Lian et al., 2026). This high proportion of intronless genes is not unique to the GRAS family and has also been reported in other gene families, including DEAD-box RNA helicases, SAUR, PPR, and F-box gene families (Aubourg et al., 1999; Jain et al., 2006; Dong et al., 2023). It has been proposed that intronless or intron-poor genes may enable more rapid transcriptional responses under stress conditions (Jeffares et al., 2008; Liu et al., 2021a). Further analysis revealed structural differences among subfamilies, which may provide the structural basis for their functional diversification. For example, DELLA subfamily members exhibit a highly conserved gene structure, uniformly containing 10 motifs arranged in the order Motif 6, 4, 1, 9, 7, 10, 8, 3, 2, 5, all possessing UTR and lacking introns. In contrast, all SCR subfamily members lack Motif 10 and contain a single intron, while all Os4 subfamily members are intronless (**Figure 4**).

Among the 38 *PqGRAS* genes responsive to cold stress in *P. quinquefolius*, spanning 10 subfamilies, the PAT1 subfamily contributed the greatest number of members (10) (**Figure 7A**; **Supplementary Table 7**), consistent with reports of widespread PAT1 subfamily involvement in cold stress responses in *A. comosus* and *P. edulis* (Lin et al., 2024; Cai et al., 2025). Furthermore, 10 *PqGRAS* genes were co-responsive to cold, ABA, and MeJA treatments, with the majority enriched in the PAT1 (4) and DELLA (3) subfamilies, suggesting that these two subfamilies may serve as central hubs for integrating multiple signaling inputs in *P. quinquefolius*. Further analysis showed that *PqGRAS086* (PAT1 subfamily) was upregulated under all three treatments (cold, ABA, and MeJA) (**Supplementary Figure 2**; **Supplementary Table 7**), indicating that its participation in the cold stress response may be linked to hormonal signaling. To investigate its potential functional mechanism, *P. quinquefolius* PAT1 subfamily members were clustered together with previously reported cold stress-related GRAS TFs. The results showed that *PqGRAS086* clustered in the same clade as *VaPAT1*, *PtrPAT1*, and *ZjCIGR1* (**Supplementary Figure 1**). Notably, all three of these TFs have been demonstrated to function as positive regulators of cold tolerance, albeit through distinct mechanisms: *VaPAT1* enhances cold tolerance through modulation of JA biosynthesis, *PtrPAT1* promotes cold tolerance via accumulation of GB, and *ZjCIGR1* confers cold tolerance potentially through transcriptional regulation of downstream *COR* genes. This phylogenetic relationship therefore suggests that *PqGRAS086* may play an analogous role in enhancing cold tolerance in *P. quinquefolius* (Kim et al., 2020; Wang et al., 2021; Ming et al., 2025). Exogenous MeJA treatment appeared to enhance cold tolerance in *P. quinquefolius* seedlings (**Figure 8A**), and RT-qPCR analysis showed that MeJA pretreatment further elevated *PqGRAS086* expression at 24 h of cold stress compared with the CK+Cold group (**Figure 8B**). Although no low-temperature responsiveness CREs (LTR elements) were detected within the 2,000 bp upstream promoter region of *PqGRAS086*, multiple MeJA-responsive CREs were identified in the same region, suggesting that *PqGRAS086* may respond to cold stress indirectly through the JA signaling pathway rather than through direct perception of low-temperature signals. Notably, the expression dynamics of *PqGRAS086* differed between the MeJA-only treatment (**Figure 7**) and the combined MeJA pretreatment and cold stress condition (**Figure 8**). Under MeJA treatment alone, *PqGRAS086* showed a transient response, peaking between 2 and 12 h and declining at 24 h, whereas under the combined treatment, the expression peak was observed at 24 h. This difference may be attributable to the distinct treatment conditions applied in the two experiments, as the additional cold stress signal in **Figure 8** may have influenced the expression dynamics of *PqGRAS086* in a manner that differs from MeJA treatment alone. However, it should be noted that the functional roles proposed in this study are primarily based on transcriptomic profiling and bioinformatic analyses. Future investigations employing overexpression, knockout, or gene silencing approaches, combined with chromatin immunoprecipitation and yeast one-hybrid assays, will be necessary to validate the precise regulatory mechanisms by which PqGRAS086 mediates cold stress adaptation in *P. quinquefolius*.

## 5 Conclusions

In the present study, a genome-wide identification and systematic analysis of the GRAS transcription factor family was performed for the first time in *P. quinquefolius*, encompassing 123 members phylogenetically resolved into 13 subfamilies. Expression profiling revealed significant tissue-specific expression patterns and differential responses to cold, MeJA, and ABA treatments among family members, with the PAT1 and DELLA subfamilies identified as key hubs responding to multiple stress signals. Furthermore, integrated phylogenetic analysis, expression profiling, and RT-qPCR identified *PqGRAS086* as a candidate gene potentially involved in the cold stress response of *P. quinquefolius* through an indirect mechanism mediated by the JA signaling pathway. Collectively, these findings advance our mechanistic understanding of GRAS-associated cold stress regulation in *P. quinquefolius* and offer promising candidate genes for the development of cold-tolerant cultivars through molecular breeding.

## Data Availability

The original contributions presented in the study are included in the article/**Supplementary Material**, further inquiries can be directed to the corresponding author/s.
