# Supplementary material for: Identification and characterization of GRAS transcription factors in Panax quinquefolius and their potential roles in cold tolerance
Source: Front Plant Sci. 2026 May 20;17:1859382. doi: 10.3389/fpls.2026.1859382 (PMC13230195; doi:10.3389/fpls.2026.1859382)
Supplement: Supplementary file 1 [file Table1.docx]

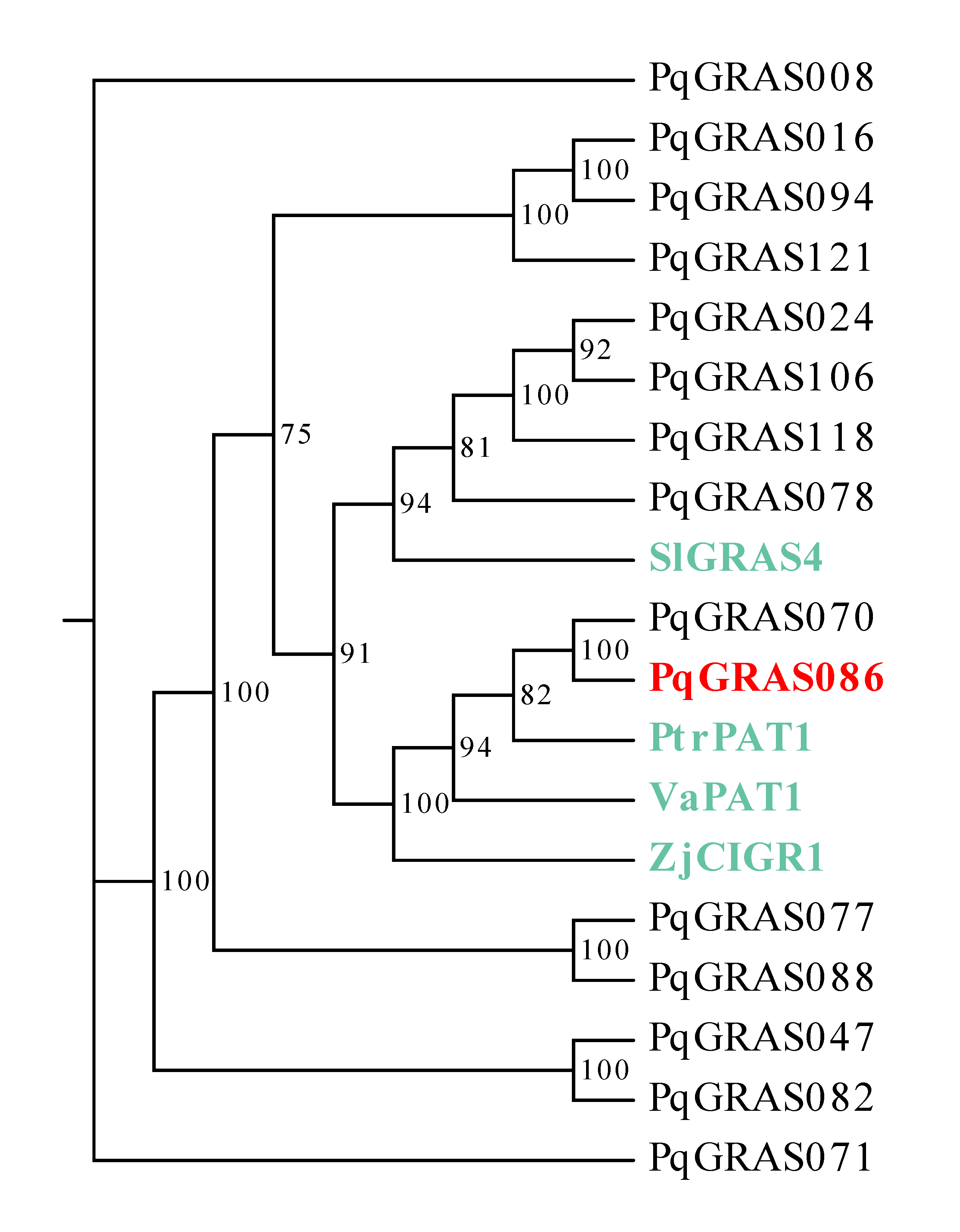


**Supplementary Figure S1.** Phylogenetic tree of PAT1 subfamily proteins in *Panax quinquefolius* with four reported cold-response proteins VaPAT1, PtrPAT1, ZjCIGR1 and SlGRAS4. The reported VaPAT1, PtrPAT1, ZjCIGR1 and SlGRAS4 were highlighted with green color, and PqGRAS086 was highlighted with red color.


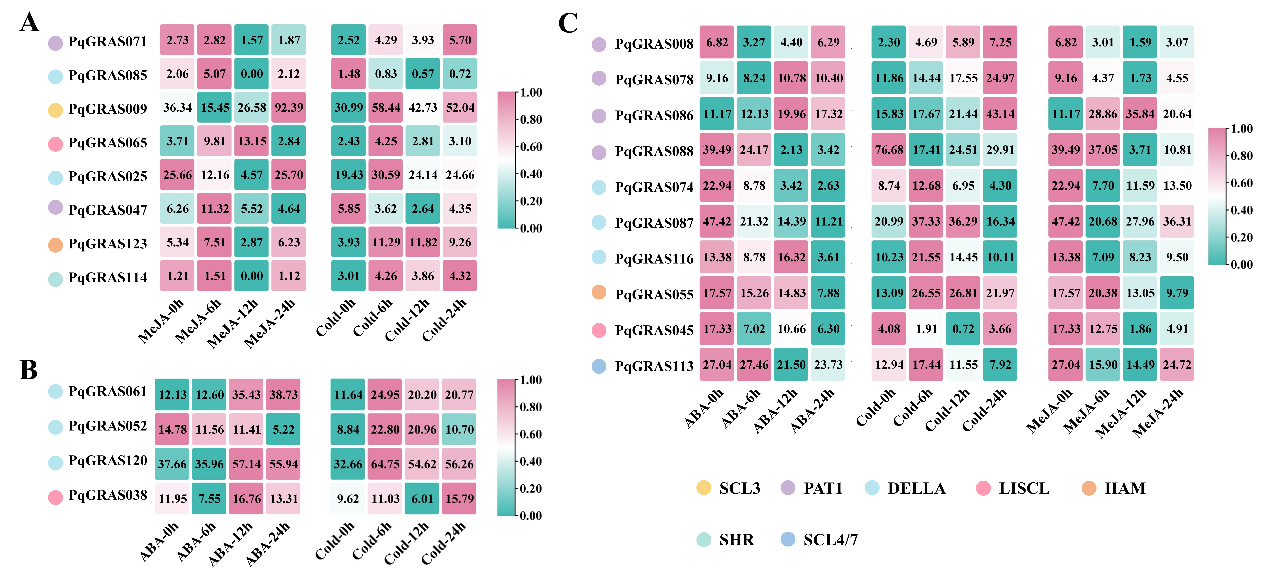


**Supplementary Figure S2.** Heatmaps of *PqGRAS* genes simultaneously responding to cold and MeJA, cold and ABA, as well as all three treatments (cold, ABA, and MeJA). (A) Heatmap of *PqGRAS* genes simultaneously responding to MeJA and cold stress. (B) Heatmap of *PqGRAS* genes simultaneously responding to ABA and cold stress. (C) Heatmap of *PqGRAS* genes simultaneously responding to ABA, MeJA and cold stress. Members of different subfamilies are marked with circles of different colors. The expression levels were normalized by row using Zero To One algorithm. The color scale at the right of the heatmap refers to the relative expression level, and the color gradient from green to pink represents increasing expression level.


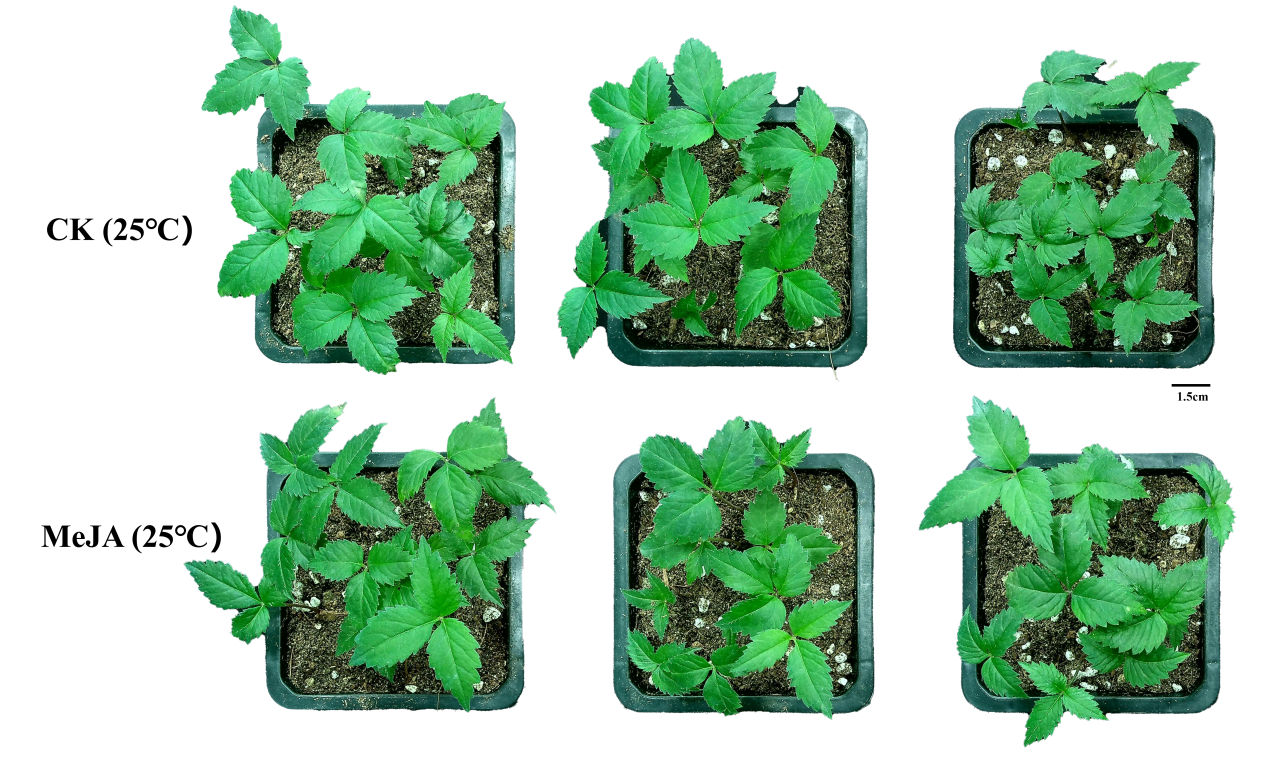


**Supplementary Figure S3.** Morphology of *P. quinquefolius* seedlings treated with MeJA (MeJA) or without MeJA (CK) at 25°C for 48 h. Bar = 1.5cm.
